# Rare-Variant Genome-Wide Association and Polygenic Score Assessment of Vitamin D Status in a Middle Eastern Population

**DOI:** 10.3390/ijms26199481

**Published:** 2025-09-28

**Authors:** Nagham Nafiz Hendi, Umm-Kulthum Umlai, Omar Albagha, Georges Nemer

**Affiliations:** 1 Faculty of Pharmacy, Applied Science University, Amman P.O. Box 11937, Jordan; n_hendi@asu.edu.jo; 2 College of Health and Life Sciences, Hamad Bin Khalifa University, Qatar Foundation, Doha P.O. Box 34110, Qatar; uiumlai@hbku.edu.qa; 3 Diabetes Research Center, Qatar Biomedical Research Institute, Hamad Bin Khalifa University, Qatar Foundation, Doha P.O. Box 34110, Qatar; 4 Department of Biochemistry and Molecular Genetics, American University of Beirut, Beirut P.O. Box 110236, Lebanon

**Keywords:** vitamin D deficiency, endocrinology, GWAS, rare variants, polygenic score, Middle Eastern genetics

## Abstract

Vitamin D deficiency is highly prevalent in the Middle East despite abundant sunlight; however, most genetic studies have focused on common variants in Europeans only. We analyzed whole-genome sequences from 13,808 Qatar Biobank participants, evaluating rare variants (minor allele frequency 0.01–0.0001) for associations with serum 25-hydroxyvitamin D (25(OH)D) levels and deficiency risk (≤20 ng/mL) in independent discovery (*n* = 5885) and replication (*n* = 7767) cohorts, followed by meta-analyses. In quantitative analyses, the discovery cohort identified 41 genome-wide significant signals, including *CD36* rs192198195 (*p* = 2.48 × 10^−8^), and replication found 46, including *SLC16A7* rs889439631 (*p* = 2.19 × 10^−8^), implicating lipid metabolism pathways. In binary analyses, replication revealed *POTEB3* rs2605913 (*p* = 2.8 × 10^−8^), while meta-analysis (*n* = 13,652) uncovered *SLC25A37* rs952825245 (*p* = 5.15 × 10^−12^), a locus associated with cancer and vitamin D signaling. Rare-variant polygenic scores derived from discovery significantly predicted continuous (R^2^ = 0.146, *p* = 9.08 × 10^−12^) and binary traits (AUC = 0.548, OR = 0.99, *p* = 9.22 × 10^−6^) in replication. This first rare-variant GWAS of vitamin D in Middle Easterners identifies novel loci and pathways, underscores the contribution of ancestry-specific rare alleles, and supports integrating rare and common variants to guide precision management in high-burden populations.

## 1. Introduction

Vitamin D plays a crucial role in maintaining calcium homeostasis, promoting skeletal health, and supporting various physiological functions. Deficiency in serum 25-hydroxyvitamin D (25(OH)D)—defined as levels below 20 ng/mL (50 nmol/L)—is a widespread global health concern. It has been linked to increased risks of osteoporosis, cardiovascular disease, immune dysfunction, and cancer [1]. Paradoxically, despite year-round sunlight, vitamin D deficiency remains alarmingly prevalent in Qatar and the wider Middle East, underscoring a multifaceted interplay of environmental, behavioral, and genetic factors [2,3].

Large genome-wide association studies (GWAS) in predominantly European populations (e.g., UK Biobank) and in regional cohorts like the Qatar Biobank (QBB) have identified several loci associated with serum 25(OH)D concentrations, like *GC* (Group-specific component, encodes vitamin D-binding protein), *CYP27A1* (encodes 27-hydroxylase), *DHCR7* (encodes 7-dehydrocholesterol reductase), and *SDR42E1* (encodes short-chain dehydrogenase/reductase 42E1) [2,4,5]. However, many of these identified variants are common (minor allele frequency, MAF > 5%), and collectively explain only a modest portion of the trait’s heritability. This leaves a significant proportion of the genetic contribution to vitamin D status unaccounted for—often referred to as the “missing heritability” [6]. Rare variants (MAF ≤ 1%) have largely been overlooked in prior GWAS; however, population genetics theory suggests they may play a pivotal role, particularly due to their potential deleterious effects maintained through purifying selection [7].

**Figure 1:**
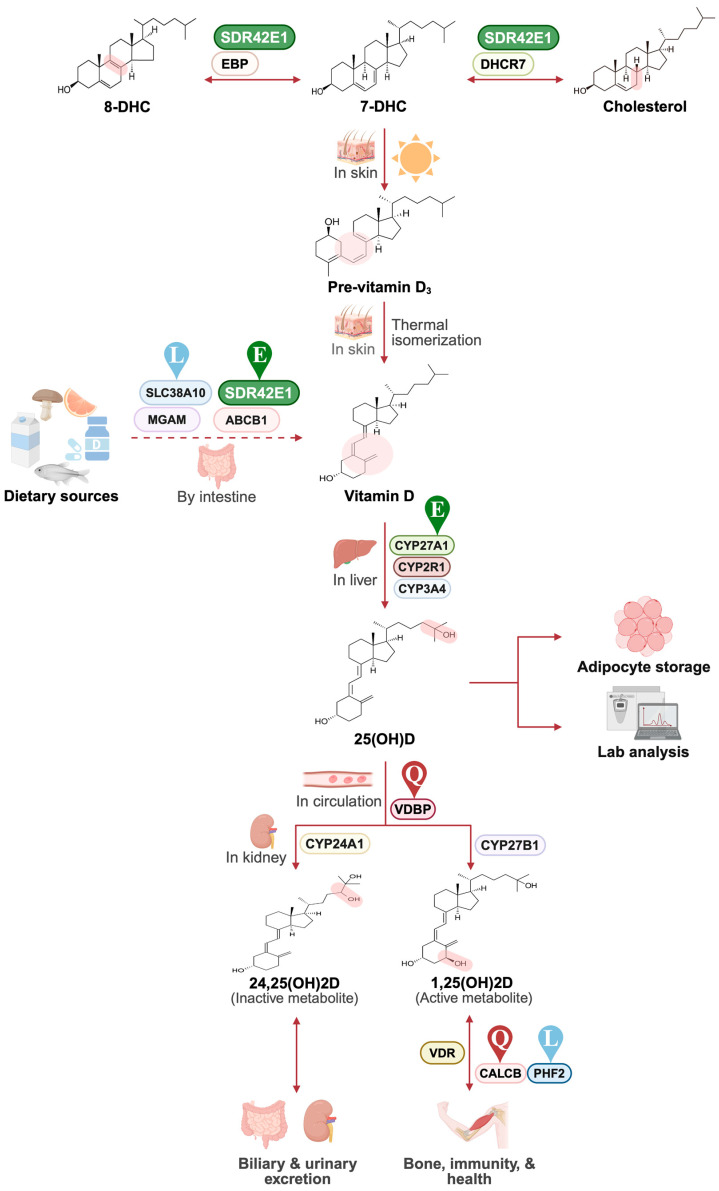
Key genetic contributors in vitamin D synthesis and metabolism. 7-dehydrocholesterol (7-DHC), the precursor of vitamin D_3_, is generated from cholesterol or 8-dehydrocholesterol (8-DHC) via EBP (Emopamil-binding protein), DHCR7 (7-dehydrocholesterol reductase), and SDR42E1 (short-chain dehydrogenase/reductase 42E1), previously reported in European cohorts (E, green drop) [4,5]. Vitamin D can also be absorbed through the intestine, with additional contributors, such as MGAM (Maltase-Glucoamylase) and SLC38A10 (Solute Carrier 38A10) identified in Lebanese cohorts (L, blue drop). In the Qatari cohort, population-specific regulation was observed through *GC* (encoding vitamin D-binding protein, VDBP) (Q, red drop). Further regulatory influences, like CALCB (Calcitonin B) and PHF2 (Plant Homeodomain Finger 2), influence vitamin D receptor (VDR) expression in osteocytes in the Qatari and Lebanese cohorts [2,3]. Chemical structures were obtained from PubChem “https://pubchem.ncbi.nlm.nih.gov (accessed on 30 May 2025)”. Created with BioRender.com by Nagham Nafiz Hendi (2025) “https://app.BioRender.com//illustrations/68cb4ff1b668a8b57e6f8f28”.

Recent studies have shown that rare, high-impact variants can substantially influence human traits and disease risk, including Mendelian disorders and monogenic forms of common diseases [8]. For example, exome sequencing has revealed rare non-synonymous and putative loss-of-function variants in *CYP2R1*—a key vitamin D 25-hydroxylase—linked to altered vitamin D metabolism and increased susceptibility to rickets [9]. Similarly, rare variants in genes such as *AGO4* (Argonaute Component 4) and ATP-related pathways have been implicated in vitamin D regulation in Korean and European cohorts [10,11]. However, such findings remain underexplored in Middle Eastern populations, which possess distinct demographic histories and genetic structures. To contextualize this rationale, Figure 1 illustrates key players in vitamin D synthesis and metabolism, highlighting canonical pathways and population-specific regulatory factors relevant to this study.

Whole-genome sequencing (WGS) offers a powerful tool to uncover population-specific rare variants that may be absent or underrepresented in global reference panels [2]. Leveraging the deeply characterized QBB cohort, this study aims to perform a large-scale rare variant association analysis targeting variants with MAF between 0.01 and 0.0001. By integrating quantitative 25(OH)D phenotypes and replicating key findings within the QBB dataset, we aim to identify novel rare variants that contribute to vitamin D levels and risk of deficiency in the Qatari population. Given that genetics explain up to 50% of 25(OH)D variability [4], this study will clarify the genetic basis of vitamin D regulation and support precision health approaches tailored to Middle Eastern populations and their disease burden, including cancer.

## 2. Results

### 2.1. Characteristics of Study Population

**Figure 2:**
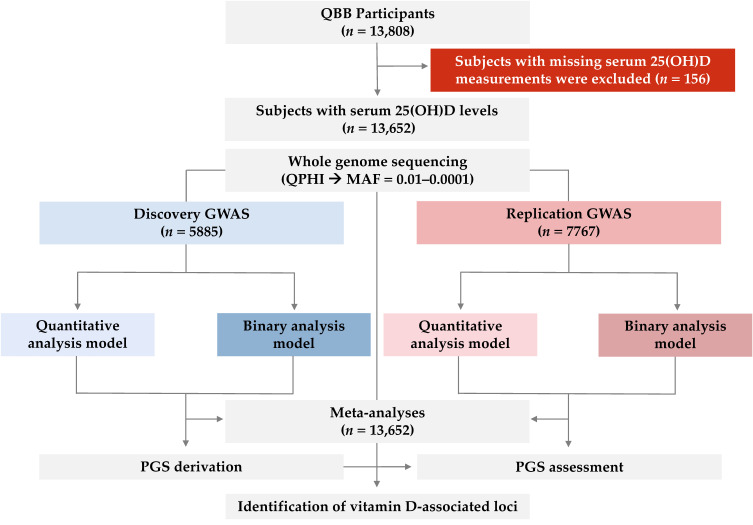
Study design for rare-variant genome-wide association analysis of vitamin D. Data were obtained from 13,808 Qatar Biobank (QBB) participants, of whom 13,652 had available serum vitamin D measurements. Vitamin D deficiency was defined as serum 25-hydroxyvitamin D (25(OH)D) levels ≤ 20 ng/mL. Whole-genome sequencing (WGS) was performed through the Qatar Precision Health Institute (QPHI). Genome-wide association studies (GWAS) were conducted separately in a discovery cohort (*n* = 5885 of 6013) and a replication cohort (*n* = 7767 of 7795) using SAIGE (Scalable and Accurate Implementation of GEneralized mixed model), under both quantitative (inverse-normal-transformed 25(OH)D) and binary (deficient vs. sufficient) models. Results were combined in fixed-effects meta-analysis (*n* = 13,652) using PLINK. Polygenic scores (PGS) were derived from discovery GWAS results and evaluated in the replication cohort.

An overview of the study design is presented in Figure 2. The overall mean age of participants was 40.1 years (±13.1), with similar age distributions across both groups. Females constituted the majority of the cohort (55.5%), while males made up 44.5%, with no significant difference (*p* = 0.075). The average body mass index (BMI) across the entire cohort was 29.6 kg/m^2^ (±6.1), placing most individuals in the overweight or obese range (BMI ≥ 25). The mean serum 25(OH)D concentration was 19.5 ng/mL (±11.1), with no difference between cohorts (*p* = 0.399). More than 60% of participants were classified as vitamin D deficient, 26.8% insufficient (20–30 ng/mL), with only 12.8% having levels in the normal range. Participant demographics and phenotype distributions are described in Table 1.

### 2.2. Rare Variants Associated with 25(OH)D Levels Identified in Quantitative Trait GWAS

**Figure 3:**
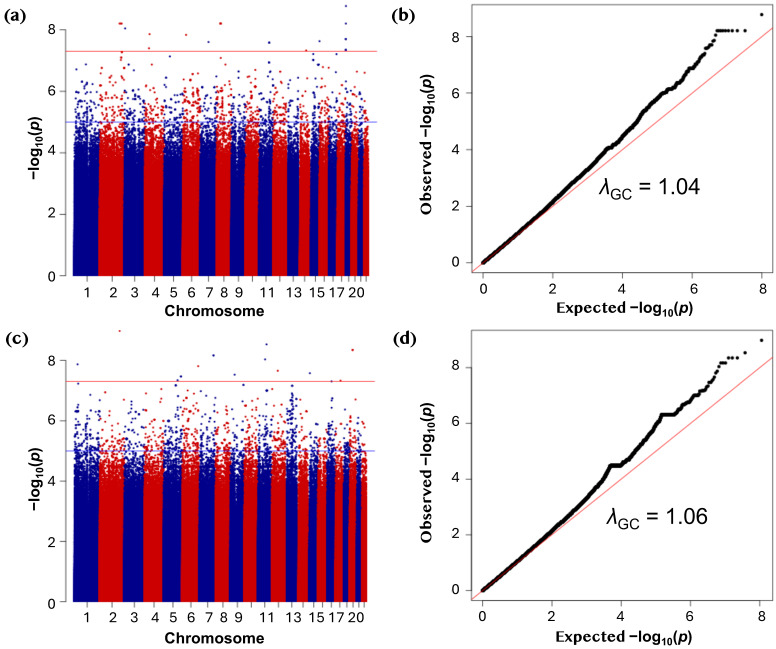
Genome-wide association analyses of serum 25(OH)D levels in discovery and replication cohorts. (**a**) Manhattan plot of GWAS results for the discovery cohort (autosomes only) using variants with minor allele frequency (MAF) between 0.0001 and 0.1. Serum 25(OH)D concentrations were inverse-normal transformed for analysis. The red and blue colors are used alternately to distinguish chromosomes. The red horizontal line denotes the genome-wide significance threshold (*p* ≤ 5.0 × 10^−8^). (**b**) Quantile–quantile (Q–Q) plot for the discovery cohort GWAS showing minimal genomic inflation (λ_GC_ = 1.04). (**c**) Manhattan plot of GWAS results for the replication cohort (autosomes only) applying the same filtering and transformation as in the discovery cohort. The red horizontal line denotes the genome-wide significance threshold (*p* ≤ 5.0 × 10^−8^). (**d**) Q–Q plot for the replication cohort GWAS showing minimal genomic inflation (λ_GC_ = 1.06).

GWAS analysis was conducted to test the association of 49,260,959 rare single-nucleotide polymorphisms (SNPs, MAF 0.0001 to 0.01) with inverse-normal-transformed 25(OH)D concentrations in the discovery dataset (*n* = 5885 participants). The results of the discovery GWAS are presented as Manhattan and Quantile–quantile (Q–Q) plots (Figure 3). The distribution of observed versus expected *p*-values demonstrated minimal deviation under the null, with a genomic inflation factor (*λ*_GC_) of 1.04 (Figure 3a), indicating appropriate control for confounding.

In total, 41 rare variants reached genome-wide significance (*p* ≤ 5.0 × 10^−8^) in the discovery phase (Figure 3b). Several of the top discovery-stage associations were rare variants located in genes implicated in lipid metabolism and vitamin D transport (Table S1). These included rs374799245 near *NFIC* (Nuclear Factor I C, effect size (*β)* = −3.42 (standard error (SE) = 0.57), *p* = 1.67 × 10^−9^), rs758713488 in *TMPRSS9* (Transmembrane Serine Protease 9, *β* = −4.04 (0.69), *p* = 6.21 × 10^−9^), and rs192198195 in *CD36* (Cluster of Differentiation 36, *β* = −1.94 (0.35), *p* = 2.48 × 10^−8^). Allele frequency comparisons indicated that most rare variants were comparable across populations (Table S2). However, several displayed marked differences: rs201217684 was observed at 0.53% in Qatar Genome Program (QGP) versus ~21–25% in Allele Frequency Aggregator (ALFA), 1000 genomes, and Genome Aggregation Database (gnomAD) datasets, rs192198195 at 0.07% in QGP versus 0.30–0.42% in 1000 Genomes and gnomAD, and rs866413364 at 0.025% in QGP but enriched in the gnomAD Middle Eastern subset (0.5%).

These findings were validated in an independent replication cohort (*n* = 7767 participants) comprising 56,489,881 rare variants analyzed using identical model specifications. Replication analysis yielded 46 genome-wide significant associations (Table S3), also with minimal genomic inflation (*λ*_GC_ = 1.06; Figure 3c,d). One of the top signals was rs889439631 in *SLC16A7* (solute carrier family 16A7; *β* = −2.69, (0.48), *p* = 2.19 × 10^−8^) and chr11:87081213:G:T in *TMEM135* (Transmembrane Protein 135; *β* = −3.62, (0.68), *p* = 9.86 × 10^−8^). Allele frequency comparisons showed that most rare variants were broadly consistent across populations (Table S2), although a few demonstrated modest differences. In particular, rs145483580 was notably rarer in QGP (0.013%) compared with reference datasets (0.15–0.25%), whereas rs184301716 and rs566089596 differed only marginally.

**Figure 4:**
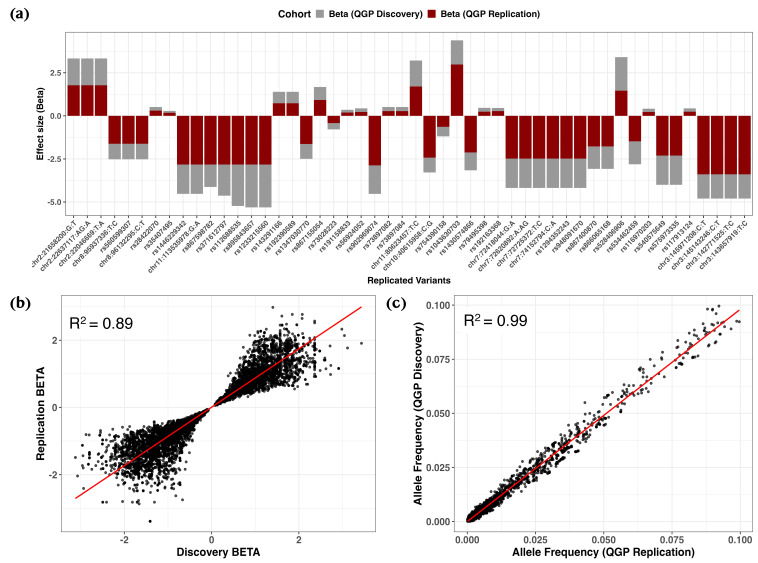
Replication of rare variant associations for serum 25(OH)D levels. (**a**) Bar plots comparing effect estimates (*β*) between discovery (gray) and replication (dark red) cohorts, highlighting variants meeting a suggestive significance threshold (*p* ≤ 3.0 × 10^−5^) with consistent effect direction. (**b**) Scatter plot of *β* estimates between cohorts, with the regression line shown in red and R^2^ indicating the coefficient of determination. (**c**) Scatter plot of allele frequencies between cohorts with corresponding regression statistics. Displayed variants met the nominal significance threshold (*p* < 0.05) and had a consistent direction of effect.

Notably, cross-cohort comparison demonstrated strong concordance, with 9192 out of 15,857 variants (58.0%) demonstrating a consistent direction of effect at nominal significance (*p* < 0.05) within a ±250 kb window between the discovery and replication cohorts (Figure 4a and Table S4). Effect sizes for the top variants (*p* ≤ 4.0 × 10^−5^) showed strong agreement between cohorts, as illustrated by a high correlation in effect weights (coefficient of determination (R^2^) = 0.869; regression slope = 0.869, 95% confidence intervals (CI): 0.863–0.876, *p* < 2.2 × 10^−16^) (Figure 4b). Similarly, allele frequencies were highly correlated between discovery and replication samples (R^2^ = 0.988; regression slope = 1.008, 95% CI: 1.006–1.010, *p* < 2.2 × 10^−16^; Figure 4c).

A fixed-effects inverse-variance meta-analysis was conducted, combining results from the discovery (*n* = 5885) and replication (*n* = 7767) cohorts for a total of 13,652 participants. Of the 374 variants carried forward for joint evaluation, 54 rare variants reached genome-wide significance, despite showing only suggestive evidence of association (*p* ≤ 5.0 × 10^−5^) in the individual datasets, with minimal between-study heterogeneity (Cochran’s Q–test *p*–heterogeneity > 0.05). Prominent genome-wide significant meta-analysis signals were observed in *CNTN3* (1-Contactin 3; rs115651661, *β* = 0.73 (0.12), *p* = 1.48 × 10^−8^) and *EBF1* (Early B-cell Factor 1; rs536115678, *β* = −1.74 (0.48), *p* = 1.57 × 10^−8^) (Table 2). A complete summary of meta-analysis statistics is provided in Table S5.

### 2.3. Rare Variant Associated with Vitamin D Deficiency Identified in Binary Trait GWAS

**Figure 5:**
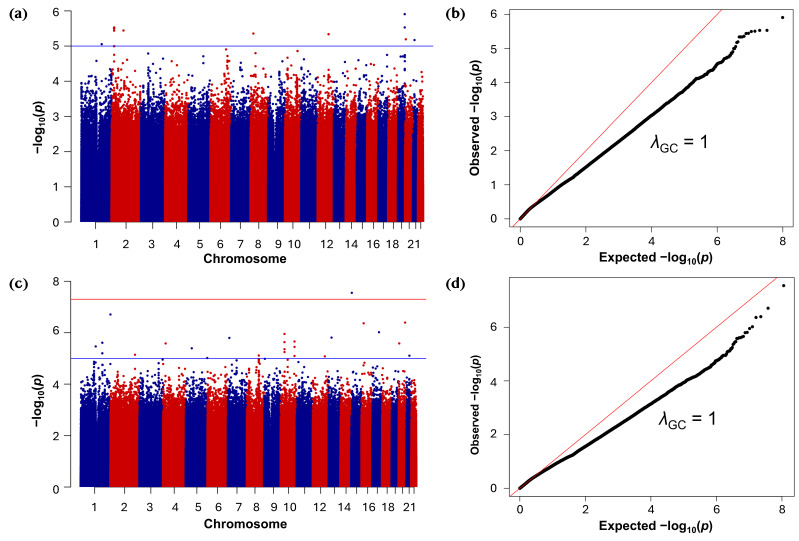
Genome-wide association analyses of vitamin D deficiency in discovery and replication cohorts. (**a**) Manhattan plot of GWAS results for the discovery cohort (autosomes only) filtered for variants with MAF 0.0001–0.1, assessing the binary vitamin D deficiency trait. The red and blue colors are used alternately to distinguish chromosomes. The blue horizontal line denotes the genome-wide suggestive significance threshold (*p* ≤ 5.0 × 10^−5^). (**b**) Quantile–quantile (Q–Q) plot for the discovery GWAS, indicating minimal genomic inflation (*λ*_GC_ = 1.00). (**c**) Manhattan plot of GWAS results for the replication cohort (autosomes only) filtered for variants with MAF 0.0001–0.1, assessing the binary vitamin D deficiency trait. The red horizontal line denotes the genome-wide significance threshold (*p* ≤ 5.0 × 10^−8^). (**d**) Q–Q plot for the replication GWAS, indicating minimal genomic inflation (*λ*_GC_ = 1.00).

To assess the contribution of rare variants (MAF 0.0001–0.01) to the risk of vitamin D deficiency, we performed a binary genome-wide association analysis using SAIGE logistic mixed models in the discovery cohort (2287 controls; 3598 cases), testing 49,260,796 variants while adjusting for age, sex, and population structure. No variants reached genome-wide significance, and the test statistics were well-calibrated (*λ*_GC_ = 1.00; Figure 5a). Nonetheless, 95 rare variants exhibited suggestive associations (*p* ≤ 5.0 × 10^−5^; Table S6 and Figure 5b).

These variants were subsequently evaluated in the replication cohort (3126 controls; 4641 cases) using the identical model specifications, testing 56,600,172 variants and demonstrating strong genomic control (*λ*_GC_ = 1.00; Figure 5c). One variant, rs2605913 in *POTEB3* (POTE Ankyrin Domain B3), exceeded genome-wide significance (*p* = 2.8 × 10^−8^, OR = 0.38 (0.07), and an additional 115 variants reached suggestive significance (Figure 5d and Table S7). Comparison of allele frequencies revealed that rs2605913 was present at 8.8% in the QGP cohort but occurred more frequently in the European dataset of 1000 Genomes (34.4%) (Table S2).

**Figure 6:**
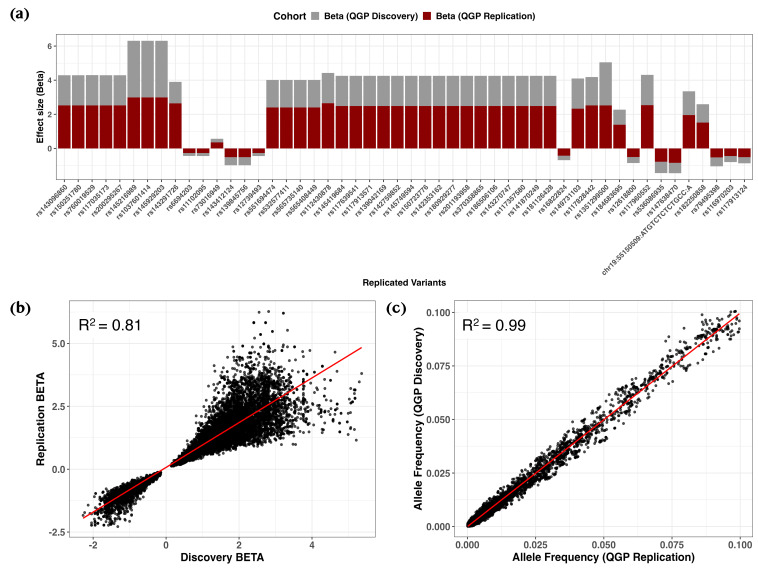
Replication analysis of rare variant associations for a binary trait. (**a**) Bar plots comparing effect estimates (odds ratios, OR) between discovery (gray) and replication cohorts (dark red), highlighting variants meeting a suggestive significance threshold (*p* ≤ 3.0 × 10^−5^) with consistent effect direction. (**b**) Scatter plot of OR between cohorts, with the red line indicating the best-fit linear regression. (**c**) Scatter plot of allele frequencies between cohorts, with the red line representing the regression fit. R^2^ denotes the coefficient of determination from correlation analysis, and 95% confidence intervals (CI) are shown for regression slopes. All displayed variants met the nominal significance threshold (*p* < 0.05) and had a consistent direction of effect.

Of the 24,503 overlapping variants, 14,866 (60.6%) showed consistent effect directions at nominal significance (Figure 6a and Table S8). Effect estimates between cohorts were highly concordant (R^2^ = 0.805, slope = 0.889; 95% CI: 0.882–0.896; *p* < 2.2 × 10^−16^; Figure 6b), and allele frequencies were closely aligned (R^2^ = 0.991, slope = 0.9936; 95% CI: 0.992–0.995; Figure 6c).

Fixed-effects Meta-analysis of the binary deficiency GWAS across the discovery and replication cohorts, comprising 13,652 participants, strengthened statistical power beyond the individual datasets. All 93 evaluated variants surpassed genome-wide significance threshold, with no evidence of heterogeneity between cohorts. These variants included signals across both coding and regulatory regions, with notable mapping to biologically relevant genes, included rs140456089 in *PPP1R12C* (Protein Phosphatase 1 Regulatory Subunit 12C; odds ratios (OR) = 1.55 (0.30), *p* = 3.12 × 10^−13^), rs1268647997 near *RDH13* (Retinol Dehydrogenase 13; OR = 1.62 (0.33), *p* = 2.13 × 10^−12^), rs952825245 in *SLC25A37* (solute carrier family 25A37; OR = 2.30, (0.47), *p* = 5.15 × 10^−12^), and rs1454700296 in *NT5C2* (Cytosolic 5′-Nucleotidase II; OR = 2.15 (0.45), *p* = 6.45 × 10^−11^) (Table 3).

### 2.4. Discovery-Derived Rare Variant Polygenic Score Predicts 25(OH)D Levels and Deficiency

Using genome-wide significant and suggestive rare variants from the discovery GWAS, we constructed four polygenic scores (PGS) applying a clumping-and-thresholding approach. Each panel retained 355,852 variants (0.32% of tested SNPs) in the replication dataset (Table 4).

**Figure 7:**
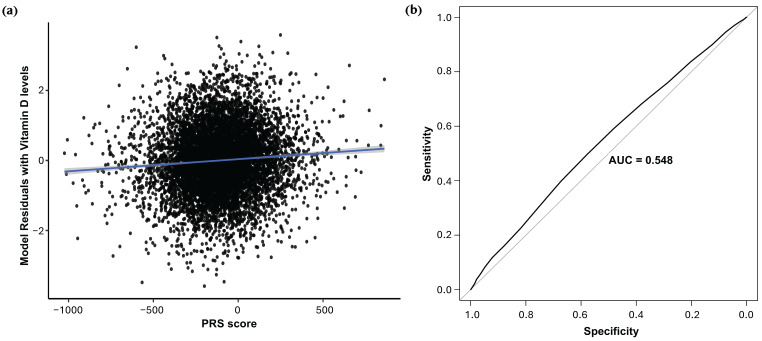
Predictive performance of discovery-derived polygenic risk scores (PRS) in the replication cohort. (**a**) Linear regression of inverse-normalized 25(OH)D levels on a representative PRS (*p* ≤ 5 × 10^−8^), adjusted for age, sex, and the first four genetic principal components. The blue line shows the linear regression line of best fit. (**b**) Receiver operating characteristic (ROC) curve for the same PRS predicting vitamin D deficiency (25(OH)D ≤ 20 ng/mL; AUC = 0.548, *p* = 9.22 × 10^−6^; OR = 0.9995; 95% CI: 0.9993–0.9997). Results for additional PRS thresholds (*p* < 5.0 × 10^−6^, *p* < 5.0 × 10^−7^, *p* < 5.0 × 10^−8^) are shown in the Supplementary Figures S1 and S2.

In linear regression adjusted for age, sex, and principal components (PCs) 1–4, all PGS demonstrated identical predictive performance for quantitative 25(OH)D levels in the replication cohort (R^2^ = 0.146, 95% CI: 0.133–0.162, *β* = 0.0004 (SE = 0.0076, *p* = 9.08 × 10^−12^) (Figure S1). Spearman correlation coefficients between the PGS and observed vitamin D levels were 0.0721 across all thresholds (*p* = 2.11 × 10^−11^, Figure 7a and Figure S2). For the binary trait (vitamin D deficiency, ≤20 ng/mL), logistic regression models yielded positive classification accuracy (AUC = 0.548, *p* = 9.22 × 10^−6^; OR = 0.9995, 95% CI: 0.9993–0.9997) across all thresholds (Figure 7b and Figure S3).

## 3. Discussion

This study presents the first GWAS in a Middle Eastern population to identify rare variants (MAF 0.01–0.0001) influencing continuous 25(OH)D concentrations and clinical vitamin D deficiency, leveraging high-coverage WGS data from over 13,000 QBB participants. The notably high prevalence of vitamin D deficiency in our cohort (>60%), consistent with earlier QBB and regional reports [2,3], underscores the public health relevance of identifying genetic determinants contributing to this trait. Despite abundant sunlight, deficiency remains widespread, a paradox attributed to a combination of cultural, lifestyle, metabolic, and genetic predisposition [1]. Most previous vitamin D GWAS have predominantly examined common variants in European cohorts [4,5], which may overlook ancestry-specific or low-frequency signals relevant to Middle Eastern populations. Our rare-variant analysis complements earlier QBB work on common-variant associations, identifying multiple genome-wide significant signals replicated across independent Qatari cohorts, thereby broadening the known genetic architecture of vitamin D and demonstrating the value of WGS in underrepresented groups.

Across quantitative and binary GWAS, we observed strong concordance of allele frequencies and effect sizes between discovery and replication cohorts, with over 60% of overlapping variants showing consistent effect directions. This reproducibility underscores the robustness of association signals across independent QBB subsets, even when individual cohort analyses lacked genome-wide significance. For binary traits, the magnitude of *β* estimates reflects the per-allele change in log-odds of deficiency; for rare variants, such effect sizes may correspond to substantial individual-level risk differences despite limited impact at the population level [6]. Mapping vitamin D deficiency as a binary trait poses inherent challenges, as dichotomizing a continuous biomarker reduces statistical power and environmental influences introduce misclassification [1,12]. The modest yield observed in single-cohort analyses in our study is therefore more likely attributable to these methodological and biological constraints than to technical bias.

Our quantitative GWAS identified 41 and 46 genome-wide significant rare variants in the discovery and replication cohorts, respectively, with several mapping to biologically plausible genes involved in lipid metabolism and nutrient transport. For example, *CD36*, a class B scavenger receptor implicated in lipid uptake, has also been linked to intestinal absorption and systemic transport of vitamin D [13], providing a potential mechanistic link to our observed associations. The rare variant rs192198195 in CD36 was associated with a 0.86-fold lower inverse-normalized 25(OH)D concentration per effect allele, potentially reducing systemic vitamin D bioavailability.

Similarly, *SLC16A7* (rs889439631) and *TMEM135* (chr11:87081213:G:T) variants were associated with an estimated ~0.95-fold reduction in vitamin D concentration per effect allele. *SLC16A7* and *TMEM135* encode membrane transporters involved in monocarboxylate and lipid handling, pathways relevant to the intracellular trafficking of lipophilic compounds like vitamin D [14,15]. These mechanisms are distinct from the canonical *GC*, *CYP2R1*, *DHCR7*, *MGAM*, and *PHF2* loci [2,3,4,5], suggesting that rare variants may capture regulatory and transport processes under stronger selective constraint. The combined meta-analysis further identified strong signals in *CNTN3* (rs115651661) and *EBF1* (rs536115678), associated with ~2.1-fold higher and ~0.18-fold lower vitamin D levels per allele, respectively. While the role in vitamin D metabolism is unclear, *EBF1* may influence vitamin D via metabolic-endocrine regulation [16], highlighting the potential impact of rare variants acting through noncanonical pathways.

In the binary deficiency analysis, no genome-wide significant variants were detected in the discovery cohort; however, replication revealed a significant association at *POTEB3* (rs2605913), with a 62% lower odds of deficiency per risk allele. *POTEB3* encodes an intracellular protein with ankyrin-repeat domains that typically mediate protein–protein interactions and cancer biology [17]. Given evidence connecting vitamin D deficiency to cancer risk [1], and that supplementation may mitigate this risk [18], the observed association may reflect regulatory pathways intersecting vitamin D and cancer biology. This novel, potentially population-specific signal warrants functional validation to clarify its role in vitamin D regulation.

Beyond the association signals, cross-population allele frequency comparisons provided additional context. While most rare variants in Qataris aligned with global datasets, some showed distinct distributions, underscoring the importance of studying ancestries that remain underrepresented in genetic research [2,3]. These differences highlight population-specific variation and underscore the need to integrate diverse cohorts to uncover novel biological mechanisms, improve the generalizability of vitamin D risk prediction models, and guide tailored prevention strategies.

Meta-analysis across cohorts substantially improved power, uncovering rare-variant associations beyond single-cohort detection—paralleling European vitamin D GWAS where low-frequency variants with larger effects complement established *GC*, *CYP2R1*, and *DHCR7* loci [2,3]. Notably, *RDH13*, encoding mitochondrial retinol dehydrogenase, connects retinoid metabolism to the VDR-retinoid X receptor (RXR) transcriptional complex, a core regulator of vitamin D-responsive gene expression [19]. Additionally, rs952825245 in *SLC25A37* (Mitoferrin-1), a mitochondrial iron importer, was associated with ≥50% higher odds of deficiency per allele. Given iron’s role in immune and endocrine function, and reported interplay with vitamin D metabolism [20], this association may reflect an indirect, biologically relevant mechanism.

Although several PGS for vitamin D have been developed from common-variant GWAS in European cohorts [5], no published PGS currently incorporates rare variants. In our previous work, we evaluated the performance of a European-derived PRS [21], which demonstrated markedly reduced predictive performance in Qataris (R^2^ = 0.098) compared to the R^2^ ≈ 0.46 reported in Europeans, and achieved only modest discrimination for vitamin D deficiency [2]. Similar findings were observed in our Lebanese cohort, where European-derived PRS performance was also diminished [3]. In both cases, predictive power was far lower than in the original European populations, underscoring the limitations of cross-ancestry portability for common-variant PRS.

In the present study, we focused exclusively on evaluating the performance of rare-variant-based PGS derived from our Qatari discovery dataset. Using genome-wide significant and suggestive rare variants from the discovery GWAS, we constructed four population-specific PRS panels, retaining over 350,000 variants in the replication dataset. Despite the low frequency of contributing alleles, these PRS explained a ~14.6% of variance in continuous 25(OH)D levels—substantially exceeding the <2% variance explained by a European-derived PRS when applied to regional data [2,3]. This improvement is consistent with broader evidence that ancestry-specific models outperform those developed in other populations, largely due to differences in linkage disequilibrium (LD) structure, allele frequency, and variant architecture [2,22]. Predictive accuracy for binary deficiency was modest (AUC = 0.55)—consistent with European PRS studies where common variants explain most variance (AUC ≈ 0.59–0.61) [21]. These findings reflect the polygenic and environmentally modifiable nature of vitamin D status, with ancestry-specific rare alleles capturing meaningful genetic risk. Integrating these rare-variant signals with common-variant predictors, and potentially with metabolic profiles, such as lipid measures relevant to vitamin D regulation, may further enhance risk stratification and support precision prevention strategies in Middle Eastern populations.

Our findings provide novel insights into the genetic architecture of vitamin D status in a large, underrepresented Middle Eastern population, yet several avenues remain for further investigation. Although seasonality was controlled by uniform sample collection, other exposures were not comprehensively captured. In Qatar, indoor lifestyles and culturally conservative clothing reduce sun exposure despite abundant sunlight; such data were not uniformly available in the QBB and could not be incorporated. Similarly, information on common medications (e.g., glucocorticoids, anticonvulsants, proton pump inhibitors, orlistat, and antiretrovirals) and comorbidities influencing vitamin D (e.g., chronic kidney or liver disease, malabsorption syndromes, obesity, and hyperparathyroidism) was not consistently available. Future efforts should explore ultra-rare variants (MAF < 0.0001), expand analyses to diverse ancestries, and incorporate environmental, clinical, and lifestyle data to refine genetic effect estimates. Functional validation of key candidates, such as *POTEB3* and *SLC25A37*, together with broader cross-ancestry gene-environment interaction studies and polygenic models integrating rare and common variants, could ultimately clarify causal mechanisms and enhance precision strategies for preventing vitamin D deficiency.

## 4. Materials and Methods

### 4.1. Study Population and Ethical Approvals

This study was conducted using data from the QBB (Doha, Qatar), a population-based prospective initiative of Qatar Foundation (QF), carried out under the Qatar Precision Health Institute (QPHI; formerly the Qatar Genome Program, QGP). Eligibility criteria included adult participants aged 18 years and older who are Qatari nationals or long-term residents (≥15 years). All participants undergo standardized clinical assessments, provide detailed lifestyle and medical history information through questionnaires, and contribute biological samples, including blood, urine, and saliva. QBB recruitment, sample handling, and data access procedures are described previously [2,23].

The current analysis focused on 13,808 Qatari individuals from the QBB cohort with high-quality WGS from the QPHI, of whom 13,652 with available serum 25(OH)D measurements were included in the final analysis. Discovery analyses were performed on 5885 participants from the first QBB data release (QGP6013), while replication analyses were based on 7767 participants from the second release (QGP7795), to allow internal validation of genetic findings within the same national cohort. Participants were recruited following ethical approval from the QBB Institutional Review Board “IRB project number QF-QGP-RES-ACC-00075 (approved on 1 January 2024)”, and all individuals gave written informed consent before participation.

### 4.2. Phenotype Measurements and Related Covariates

Serum 25(OH)D levels were quantified in the diagnostic laboratories of Hamad Medical Corporation (Doha, Qatar) using a standardized chemiluminescent immunoassay (CLIA) platform (LIAISON^®^, DiaSorin S.p.A., Saluggia, Italy). Briefly, blood samples were centrifuged for serum separation and stored at −80 °C before biochemical analysis. Full methodological details, including assay protocol and instrument calibration, have been previously described.

Two phenotype definitions were used in this study. First, for quantitative genetic analysis, raw serum 25(OH)D concentrations (in ng/mL) were normalized using rank-based inverse-normal transformation implemented in R (v3.4.0). This transformation normalizes the distribution, minimizes skewness, and reduces the influence of outliers in association models. Second, a binary vitamin D deficiency phenotype was defined using serum 25(OH)D concentrations, with individuals classified as deficient if 25(OH)D ≤ 20 ng/mL and as sufficient controls if 25(OH)D ≥ 30 ng/mL. These clinically relevant thresholds are consistent with international guidelines and have been widely adopted in nutritional epidemiology and previous vitamin D GWAS.

Anthropometric measures, including weight and height, were obtained during physical examinations using standardized equipment (Seca 284 stadiometer and balance), and BMI was calculated as weight (kg) divided by the square of height (m^2^).

Baseline demographic and phenotypic characteristics were compared between the discovery and replication cohorts using R (v3.4.0). Continuous variables were tested using Welch’s *t*-test, while categorical variables were compared using χ^2^ tests.

### 4.3. Whole-Genome Sequencing and Quality Control

The procedures for genomic DNA extraction and WGS have been detailed in a prior publication [24]. Concisely, DNA quantity and integrity were assessed using the Quant-iT dsDNA Assay Kit (Invitrogen, Thermo Fisher Scientific, Waltham, MA, USA) and FlexStation 3 reader (Molecular Devices, San Jose, CA, USA). High-coverage WGS (30× depth) was performed at Sidra Medicine Genomics Facility (Doha, Qatar) on the Illumina HiSeq X Ten (Illumina Inc., San Diego, CA, USA). Raw reads were quality-checked with FastQC (v0.11.2; Babraham Bioinformatics, Cambridge, UK), aligned to the human reference genome GRCh38 using BWA-MEM (v0.7.12), and variants were called using GATK HaplotypeCaller (v3.4; Broad Institute, Cambridge, MA, USA). Joint genotyping was conducted on consolidated gVCFs via GenomicsDB, and variants were filtered via GATK’s Variant Quality Score Recalibration (VQSR), retaining only “PASS” variants for analysis.

Stringent quality control (QC) procedures were applied at both the sample and variant level to ensure high confidence in genetic findings through PLINK (v2.0) [25]. Samples were excluded if they had genotype call rates <95%, ambiguous or mismatched gender, excess heterozygosity (>±4 standard deviations from the mean), or were identified as duplicates. Population structure was assessed using multidimensional scaling (MDS) and pairwise identity-by-state (IBS) analysis, based on a pruned set of independent autosomal SNPs selected using an LD threshold of r^2^ < 0.05 within a sliding window of 200 SNPs. Individuals deviating more than ±4 standard deviations from the first two MDS components were flagged as population outliers and excluded. Variant-level QC excluded SNPs with MAF outside the rare range of interest (i.e., <0.0001 or >0.01), genotype call rate <90%, those on the X chromosome, or those deviating from Hardy–Weinberg equilibrium (*p*-value < 1.0 × 10^−6^). The final analyses included 49,260,795 high-quality rare variants (MAF 0.0001–0.01) in the discovery dataset and 56,600,172 in the replication dataset for the binary trait, and 49,260,959 and 56,489,881 variants, respectively, for the quantitative trait, each analyzed separately.

### 4.4. Genome-Wide Association Analyses

Genome-wide association analyses were conducted using a two-stage design to identify rare genetic variants (MAF 0.0001–0.01) associated with serum 25(OH)D levels. The discovery stage included 5884 participants from the discovery cohort, while the replication stage involved 7767 independent participants from the replication cohort. Association testing was performed separately in each cohort using the SAIGE/R package (Scalable and Accurate Implementation of GEneralized mixed model, v1.5.0), a computationally efficient mixed-model regression framework that accounts for sample relatedness and case–control imbalance [26].

For the quantitative trait (rank-based inverse-normalized 25(OH)D), linear mixed models were applied; for the binary trait of vitamin D deficiency, logistic mixed models were used. All models included covariates for age, sex, and the first four genetic PCs to correct for population stratification. As all samples were collected during a comparable sunny season in Qatar, seasonality was not considered a confounding factor in the analysis. Genome-wide significance was defined as *p* < 5.0 × 10^−8^, suggestive significance as *p* < 1.0 × 10^−5^, and nominal significance as *p* < 0.05.

To evaluate replication and assess the correlation of effect sizes and allele frequencies between cohorts, we first identified loci associated with 25(OH)D that were driven by the same lead variants in both the discovery and replication datasets. We then examined variants within a ±250 kb region flanking each genome-wide significant signal from the discovery cohort to identify additional replicated associations. Variants passing genome-wide significance (*p* < 5.0 × 10^−8^) in discovery were considered replicated if they showed nominal significance (*p* < 0.05) and had a consistent direction of effect (based on beta coefficients) in the replication cohort. To assess cross-cohort concordance, we performed linear regression analyses comparing allele frequencies and effect sizes between cohorts, reporting the regression slope, 95% CI, and R^2^.

Subsequently, a fixed-effects inverse-variance-weighted meta-analysis was implemented in PLINK (v2.0) to combine results across both datasets, encompassing 13,652 participants [25]. Heterogeneity across cohorts was assessed using Cochran’s Q test [27]. The impact of associated variants was evaluated based on the magnitude and direction of their beta coefficients. Q–Q plots, Manhattan plots, and *λ*_GC_ were generated using R (v4.4.1). Our discovery dataset demonstrated sufficient statistical power (≥95%) to detect variants with an effect size of *β* = −3.03 at the genome-wide significance threshold (*p* < 5.0 × 10^−8^).

### 4.5. Polygenic Score Construction and Evaluation

PGS were constructed to estimate individual genetic predisposition to serum 25(OH)D levels based on the cumulative effect of associated rare variants. We derived the PGS using summary statistics from the discovery GWAS dataset, applying the clumping-and-thresholding (C + T) approach as implemented in PLINK (v1.9) [28]. This method identifies sets of independent variants by grouping SNPs in LD around index SNPs based on a predefined LD threshold (r^2^ = 0.2) and applying varying *p*-value cutoffs to include only statistically relevant variants.

PGS were generated across a range of *p*-value thresholds (from 5.0 × 10^−8^ to 5.0 × 10^−1^), resulting in multiple rare-variant-based PGS panels, sequentially labeled from Q-PGS1 to QGP-4 according to decreasing statistical stringency. Each PGS was tested for predictive performance in the replication cohort (QGP7795, *n* = 7767) using linear regression models for the continuous trait and logistic regression for vitamin D deficiency. Models were adjusted for age, sex, and the first four PCs to account for population structure. The optimal PGS panel was identified based on the highest adjusted R^2^ for the quantitative phenotype and AUC for the binary phenotype.

### 4.6. Variant Annotation and Functional Characterization

Genome-wide significant variants identified in GWAS and meta-analyses were annotated using the Ensembl Variant Effect Predictor (VEP; GRCh38, release 114) [29]. Functional consequences (e.g., missense, intronic, intergenic), gene proximity, and regulatory annotations were also extracted. Variant frequencies were compared against global populations using the gnomAD “https://gnomad.broadinstitute.org (accessed on 9 September 2025)”, the ALFA “www.ncbi.nlm.nih.gov/snp/docs/gsr/alfa/ (accessed on 9 September 2025)”, and the 1000 Genomes Project [30].

## 5. Conclusions

This rare-variant GWAS in a Middle Eastern population identifies novel genetic determinants of vitamin D status, implicating diverse metabolic and regulatory pathways. The findings establish an ancestry-specific genetic foundation for functional validation and precision medicine. In clinical practice, population-tailored polygenic risk models could help stratify high-burden populations, supporting earlier screening, targeted supplementation, and culturally adapted prevention strategies, particularly for patients with vitamin D-modifying conditions or therapies, where genetic risk information may guide personalized care.

## Figures and Tables

**Table 1:** Characteristics of study population.

Cohort	Discovery	Replication	All Subjects	*p*-Value
Sample size	5885	7767	13,652	-
Male *n* (%)	2567 (43.6)	3508 (45.2)	6075 (44.5)	0.0746
Female n (%)	3318 (56.4)	4259 (54.8)	7577 (55.5)	
Mean age ± SD	39.75 ± 12.83	40.38 ± 13.37	40.11 ± 13.14	0.00506
BMI (kg/m^2^)	29.38 ± 6.05	29.69 ± 6.14	29.55 ± 6.10	0.00387
Vit D (ng/mL) ± SD	19.36 ± 11.12	19.52 ± 11.14	19.45 ± 11.13	0.399
Normal Vit D *n* (%)	675 (11.5)	1073 (13.8)	1748 (12.8)	0.0000546
Insufficient Vit D *n* (%)	1612 (27.4)	2053 (26.4)	3665 (26.8)	0.218
Deficient Vit D *n* (%)	3598 (61.1)	4641 (59.8)	8239 (60.4)	0.105

Descriptive statistics of the Qatar Biobank (QBB) cohort used in the discovery (batch 1) and replication (batch 2) analyses. Continuous variables are presented as mean ± standard deviation (SD), and categorical variables are presented as number (percentage, %). *n* denotes the sample size. Vitamin D status was categorized based on serum 25-hydroxyvitamin D (25(OH)D) levels as follows: normal (>30 ng/mL), insufficient (20–30 ng/mL), and deficient (≤20 ng/mL). BMI = body mass index (kg/m^2^). The Discovery and Replication cohorts correspond to samples collected in separate batches for the genomic analysis. *p*-values reflect comparisons between Discovery and Replication cohorts (*t*-tests for continuous variables; χ^2^ tests for categorical variables).

**Table 2:** Rare variants significantly associated with vitamin D levels in a quantitative GWAS meta-analysis of the discovery and replication cohorts.

SNP	CHR	Position (BP)	Mapped Gene	HGVS ID	Consequence	A1	A2	GWAS in Replication (*n* = 7767)			GWAS in Discovery (*n* = 5885)			Meta-Analysis (*n* = 13,652)		
								MAF (A1)	Beta (SE)	*p*-Value	MAF (A1)	Beta (SE)	*p*-Value	*p*-Value	BETA	*p*-Het
rs115651661	3	74272255	*CNTN3*	NC_000003.12:g.74272255T>C	Intron	C	T	0.0040	0.48 (0.12)	0.00007	0.0027	0.73 (0.17)	2.5 × 10^−5^	1.5 × 10^−8^	0.56	0.22
rs536115678	5	158884335	*EBF1*	NC_000005.10:g.158884335C>A	Intron	A	C	0.0003	−1.84 (0.48)	0.00013	0.0003	−1.67 (0.40)	3.0 × 10^−5^	1.6 × 10^−8^	−1.74	0.79
chr21:43954055:C:T	21	43954055	*AGPAT3*	-	Intron	T	C	0.0004	1.26 (0.39)	0.00138	0.0007	1.57 (0.34)	6.1 × 10^−6^	3.7 × 10^−8^	1.43	0.55
chr21:43790823:A:G	21	43790823	*RRP1*	-	Upstream gene	G	A	0.0004	1.26 (0.39)	0.00138	0.0007	1.57 (0.34)	6.1 × 10^−6^	3.7 × 10^−8^	1.43	0.55
rs550626115	15	63022380	*TPM1-AS*	NC_000015.10:g.63022380C>T	Intron	T	C	0.0001	−2.41 (0.68)	0.00039	0.0002	−2.9 (0.69)	2.4 × 10^−5^	4.1 × 10^−8^	−2.67	0.59
rs1014490316	20	59592010	*PHACTR3*	NC_000020.11:g.59592010A>G	Intron	G	A	0.0001	2.33 (0.68)	0.00063	0.0002	2.98 (0.69)	1.7 × 10^−5^	5.0 × 10^−8^	2.65	0.50

Presented data are the same variants in QGP (Vitamin D rare GWAS) with the *p*-het (*p*-value for Cochran’s Q heterogeneity statistic) > 0.05 and *p*-value ≤ 7.0 × 10^−8^ of the meta-analysis. Please refer to Supplementary Table S1 for detailed parameter definitions.

**Table 3:** Rare variants significantly associated with vitamin D deficiency in a binary GWAS meta-analysis of the replication and discovery cohorts.

SNP	Gene Mapped	CHR	Position (BP)	HGVS ID	Consequence	A1	A2	GWAS in Replication (*n* = 7767)			GWAS in Discovery (*n* = 5885)			Meta-Analysis (*n* = 13,652)		
								MAF (A1)	Beta (SE)	*p*-Value	MAF (A1)	Beta (SE)	*p*-Value	*p*-Value	OR	*p*-Het
rs140456089	*PPP1R12C*	19	55096117	NC_000019.10:g.55096117G>A	synonymous	A	G	0.0042	1.55 (0.30)	2.6 × 10^−7^	0.0042	1.55 (0.30)	2.6 × 10^−7^	3.1 × 10^−12^	4.70	1
rs1268647997	*RDH13*	19	55065320	NC_000019.10:g.55065320G>A	upstream gene	A	G	0.0036	1.62 (0.33)	6.8 × 10^−7^	0.0830	0.36 (0.07)	6.8 × 10^−7^	2.1 × 10^−12^	5.07	1
rs62122090	*HS1BP3*	2	20628549	NC_000002.12:g.20628549C>T	intron	T	C	0.0830	0.36 (0.07)	6.8 × 10^−7^	0.0036	1.62 (0.33)	6.8 × 10^−7^	2.1 × 10^−12^	1.44	1
rs73916930	*HS1BP3*	2	20628921	NC_000002.12:g.20628921G>T	intron	T	G	0.0830	0.36 (0.07)	7.2 × 10^−7^	0.0830	0.36 (0.07)	7.2 × 10^−7^	2.4 × 10^−12^	1.44	1
rs4426492	*HS1BP3*	2	20633127	NC_000002.12:g.20633127G>A	intron	A	G	0.0823	0.36 (0.07)	7.4 × 10^−7^	0.0823	0.36 (0.07)	7.4 × 10^−7^	2.5 × 10^−12^	1.44	1
rs1185902565	*ANKRD36B*	2	97591789	NC_000002.12:g.97591790del	upstream gene	T	TC	0.0022	2.09 (0.43)	8.5 × 10^−7^	0.0022	2.09 (0.43)	8.5 × 10^−7^	3.3 × 10^−12^	8.11	1
rs73916931	*HS1BP3*	2	20628926	NC_000002.12:g.20628926G>A	intron	A	G	0.0826	0.36 (0.07)	8.6 × 10^−7^	0.0826	0.36 (0.07)	8.6 × 10^−6^	3.4 × 10^−12^	1.43	1
rs952825245	*SLC25A37*	8	23537529	NC_000008.11:g.23537529C>T	intron	T	C	0.0018	2.30 (0.47)	1.1 × 10^−6^	0.0018	2.30 (0.47)	1.1 × 10^−6^	5.2 × 10^−12^	9.95	1
rs143947667	*ATP2B1-AS1*	12	89934704	NC_000012.12:g.89934704A>G	intron, NCT	G	A	0.0060	1.24 (0.25)	1.1× 10^−6^	0.0060	1.24 (0.25)	1.1 × 10^−6^	5.7 × 10^−12^	3.45	1
rs150425221	*ATP2B1-AS1*	12	89951086	NC_000012.12:g.89951086T>C	intron, NCT	C	T	0.0060	1.24 (0.25)	1.1 × 10^−6^	0.0060	1.24 (0.25)	1.1 × 10^−6^	5.7 × 10^−12^	3.45	1
rs150021601	*ATP2B1-AS1*	12	90017542	NC_000012.12:g.90017542A>C	intron, NCT	C	A	0.0060	1.24 (0.25)	1.1 × 10^−6^	0.0060	1.24 (0.25)	1.1 × 10^−6^	5.7 × 10^−12^	3.45	1
rs143313202	*-*	20	5666007	NC_000020.11:g.5666007A>G	intergenic	G	A	0.0144	0.81 (0.17)	1.6 × 10^−6^	0.0144	0.81 (0.17)	1.6 × 10^−6^	1.2 × 10^−11^	2.25	1
rs73195003	*BTG3*	21	17610076	NC_000021.9:g.17610076A>G	intron	G	A	0.0339	−0.52 (0.11)	1.7 × 10^−6^	0.0339	−0.52 (0.11)	1.7 × 10^−6^	1.3 × 10^−11^	0.59	1
rs140599862	*-*	1	167559027	NC_000001.11:g.167559027T>C	intron, NCT	C	T	0.0032	1.71 (0.36)	2.3 × 10^−6^	0.0032	1.71 (0.36)	2.3 × 10^−6^	2.4 × 10^−11^	5.53	1
rs80111761	*HS1BP3*	2	20615440	NC_000002.12:g.20615440A>C	downstream gene	C	A	0.0374	0.50 (0.11)	2.7 × 10^−6^	0.0374	0.50 (0.11)	2.7 × 10^−6^	3.2 × 10^−11^	1.65	1
rs62125675	*HS1BP3*	2	20616068	NC_000002.12:g.20616068C>T	downstream gene	T	C	0.0374	0.50 (0.11)	2.7 × 10^−6^	0.0374	0.50 (0.11)	2.7 × 10^−6^	3.1 × 10^−11^	1.65	1
rs73776179	*LAMA2*	6	129485004	NC_000006.12:g.129485004A>G	intron	G	A	0.0049	1.39 (0.30)	3.4 × 10^−6^	0.0049	1.39 (0.30)	3.4 × 10^−6^	5.1 × 10^−11^	4.01	1
rs1454700296	*NT5C2*	10	103270756	NC_000010.11:g.103270756A>G	intron	G	A	0.0017	2.15 (0.46)	3.6 × 10^−6^	0.0017	2.15 (0.46)	3.9 × 10^−6^	6.5 × 10^−11^	8.56	1
rs1315965692	*NT5C2*	10	103270757	NC_000010.11:g.103270757G>A	intron	A	G	0.0017	2.15 (0.46)	3.9 × 10^−6^	0.0017	2.15 (0.46)	3.9 × 10^−6^	6.5 × 10^−11^	8.56	1
rs867934853	*-*	8	41159441	NC_000008.11:g.41159441G>T	downstream gene	T	G	0.0011	2.42 (0.53)	4.5 × 10^−6^	0.0011	2.42 (0.53)	4.5 × 10^−6^	8.8 × 10^−11^	11.26	1
rs563431181	*PXK*	3	58386670	NC_000003.12:g.58386670A>G	intron	G	A	0.0021	1.89 (0.41)	4.6 × 10^−6^	0.0021	1.89 (0.41)	4.6 × 10^−6^	9.2 × 10^−11^	6.64	1
rs6708069	*HS1BP3*	2	20616814	NC_000002.12:g.20616814C>A	downstream gene	A	C	0.0360	0.50 (0.11)	5.1 × 10^−6^	0.0360	0.50 (0.11)	5.1 × 10^−6^	1.1× 10^−10^	1.64	1

Please refer to Table 1 for parameter details. Abbreviations: NCT, non-coding transcript.

**Table 4:** Performance of Candidate polygenic scores (PGS) for vitamin D levels in the replication cohort.

PGS Score	PGS Name	Available Variants/Variants in Score (%)	Correlation				
			Adjusted R^2^	(95% CI)	BETA (SE)	*p*-Value	Rho
*p* < 5.0 × 10^−5^_*r*^2^ < 0.2	Q-PGS1	355,852/112,176,302 (0.32%)	0.146	(0.133–0.162)	0.0004 (0.0076)	9.1 × 10^−12^	0.0721
*p* < 5.0 × 10^−6^_*r*^2^ < 0.2	Q-PGS2	355,852/112,176,302 (0.32%)	0.146	(0.133–0.162)	0.0004 (0.0076)	9.1 × 10^−12^	0.0721
*p* < 5.0 × 10^−7^_*r*^2^ < 0.2	Q-PGS3	355,852/112,176,302 (0.32%)	0.146	(0.133–0.162)	0.0004 (0.0076)	9.1 × 10^−12^	0.0721
*p* < 5.0 × 10^−8^_*r*^2^ < 0.2	Q-PGS4	355,852/112,176,302 (0.32%)	0.146	(0.133–0.162)	0.0004 (0.0076)	9.1 × 10^−12^	0.0721

PGS, Polygenic Score; CI, Confidence interval; SE, Standard Error; Rho, Spearman correlation coefficient.

## Data Availability

The data presented in this study are available on request from the corresponding author due to licensing and access restrictions. Due to data privacy regulations, raw whole-genome sequencing data from Qatar Biobank cannot be deposited in public repositories. Access to QBB/QPHI phenotype and sequencing data is available through an ISO-certified process, requiring submission of a project application at “https://www.qphi.org.qa/research/how-to-apply (accessed on 9 January 2024)” and approval by the QBB Institutional Review Board. The GWAS summary statistics generated in this study are available in the NHGRI-EBI GWAS Catalog (https://ftp.ebi.ac.uk/pub/databases/gwas/summary_statistics/) under accession numbers GCST90667550, GCST90667551, GCST90667552, and GCST90667553. The corresponding polygenic score (PGS) data have been deposited in the PGS Catalog (https://www.pgscatalog.org) under accession number PGS005286, associated with the publication ID PGP000758.

## Supplementary Materials

The following supporting information can be downloaded at: https://www.mdpi.com/article/10.3390/ijms26199481/s1.

## Abbreviations

The following abbreviations are used in this manuscript:

25(OH)D	25-hydroxyvitamin D
AUC	Area under the curve
*AGO4*	Argonaute Component 4
BMI	Body mass index
CI	Confidence interval
CLIA	Chemiluminescent immunoassay
C + T	Clumping and thresholding
*GC*	Group-specific component (vitamin D-binding protein)
GWAS	Genome-wide association study
*DHCR7*	7-dehydrocholesterol reductase
IBS	Identity-by-state
IRB	Institutional Review Board
LD	Linkage disequilibrium
MAF	Minor allele frequency
MDS	Multidimensional scaling
OR	Odds ratio
PC	Principal component
PGS	Polygenic score
QC	Quality control
QBB	Qatar Biobank
QF	Qatar Foundation
QGP	Qatar Genome Program
QPHI	Qatar Precision Health Institute
Q–Q	Quantile–quantile
R^2^	Coefficient of determination
*POTEB3*	POTE Ankyrin Domain B3
*RDH13*	Retinol dehydrogenase 13
RXR	Retinoid X receptor
SAIGE	Scalable and Accurate Implementation of GEneralized mixed model
SE	Standard error
SNP	Single nucleotide polymorphism
*SLC*	Solute carrier
*TMEM*	Transmembrane protein
*VDR*	Vitamin D receptor
VEP	Variant Effect Predictor
WGS	Whole-genome sequencing
