# Supplementary material for: Rare-Variant Genome-Wide Association and Polygenic Score Assessment of Vitamin D Status in a Middle Eastern Population
Source: Int J Mol Sci. 2025 Sep 28;26(19):9481. doi: 10.3390/ijms26199481 (PMC12524746; doi:10.3390/ijms26199481)
Supplement: Supplementary file 1 [file ijms-26-09481-s001.zip › Supplementary Figures_Rare_GWAS.pdf]

Supplementary information

Rare-Variant Genome-Wide Association and Polygenic Score Assessment of Vitamin D Status in a Middle Eastern Population

Nagham Nafiz Hendi [1], Umm-Kulthum Umlai [2], Omar Albagha [2,3,*], Georges Nemer [2,4,*]

[1] Faculty of Pharmacy, Applied Science University, P.O. Box 11937, Amman, Jordan; naghindi93@gmail.com.
[2] College of Health and Life Sciences, Hamad Bin Khalifa University, Qatar Foundation, P.O. Box 34110, Doha, Qatar.
[3] Diabetes Research Center, Qatar Biomedical Research Institute, Hamad Bin Khalifa University, Qatar Foundation, P.O. Box 34110, Doha, Qatar.
[4] Department of Biochemistry and Molecular Genetics, American University of Beirut, P.O. Box 110236, Beirut, Lebanon.
* Correspondence: nemer@hbku.edu.qa (G.N.); oalbagha@hbku.edu.qa (O.A.).

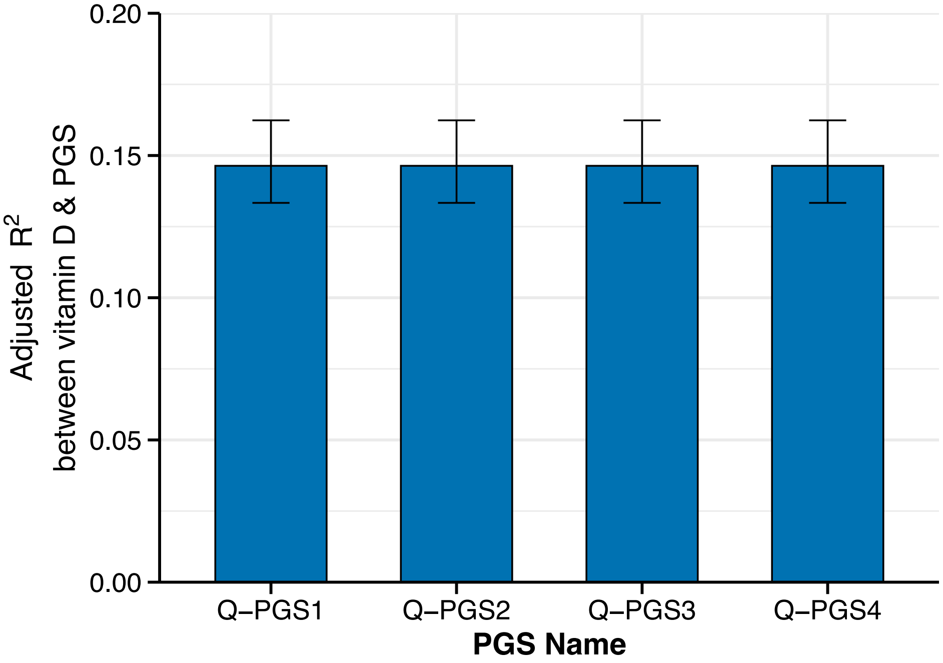

Figure S1. Predictive performance (adjusted R^2) of rare-variant polygenic scores for quantitative 25(OH)D across multiple *P*-value thresholds in the replication cohort. Bar plot showing mean adjusted R^2 values from linear regression models predicting inverse-normalized vitamin D levels using polygenic scores (PGS) constructed at varying *P*-value thresholds (x-axis) with linkage disequilibrium (LD) clumping at $r^2 < 0.2$. Models were adjusted for age, sex, and the first four genetic principal components (PC1–PC4). Error bars represent 95% confidence intervals (CIs). Blue bars indicate LD clumping at $r^2 < 0.2$. PGS thresholds: Q-PGS1 ($P < 5\times10^{-5}$), Q-PGS2 ($P < 5\times10^{-6}$), Q-PGS3 ($P < 5\times10^{-7}$), Q-PGS4 ($P < 5\times10^{-8}$).

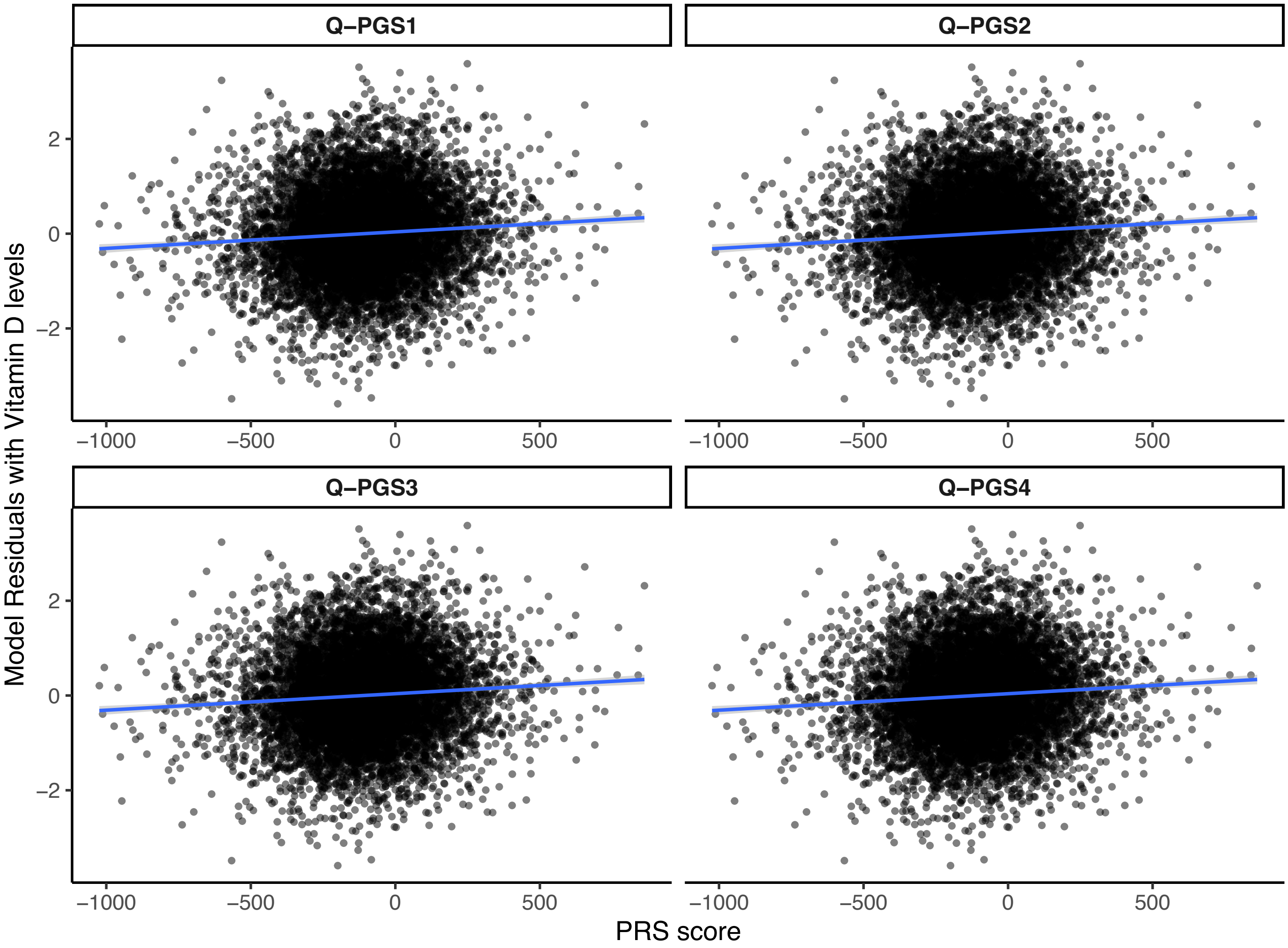

Figure S2. Linear regression of inverse-normalized baseline 25(OH)D levels on weighted polygenic risk scores (PRS) derived from a discovery dataset. It shows consistent results across all panels (R^2= 0.076, P = 2.11 × 10^{-11}). Each point represents an individual participant; the blue line indicates the fitted regression, and the shaded area shows the 95% confidence interval. Results are presented for four PRS panels constructed at different P-value thresholds: Q-PGS1 (P < $5{\times}10^{-5}$), Q-PGS2 (P < $5{\times}10^{-6}$), Q-PGS3 (P < $5{\times}10^{-7}$), and Q-PGS4 (P < $5{\times}10^{-8}$).

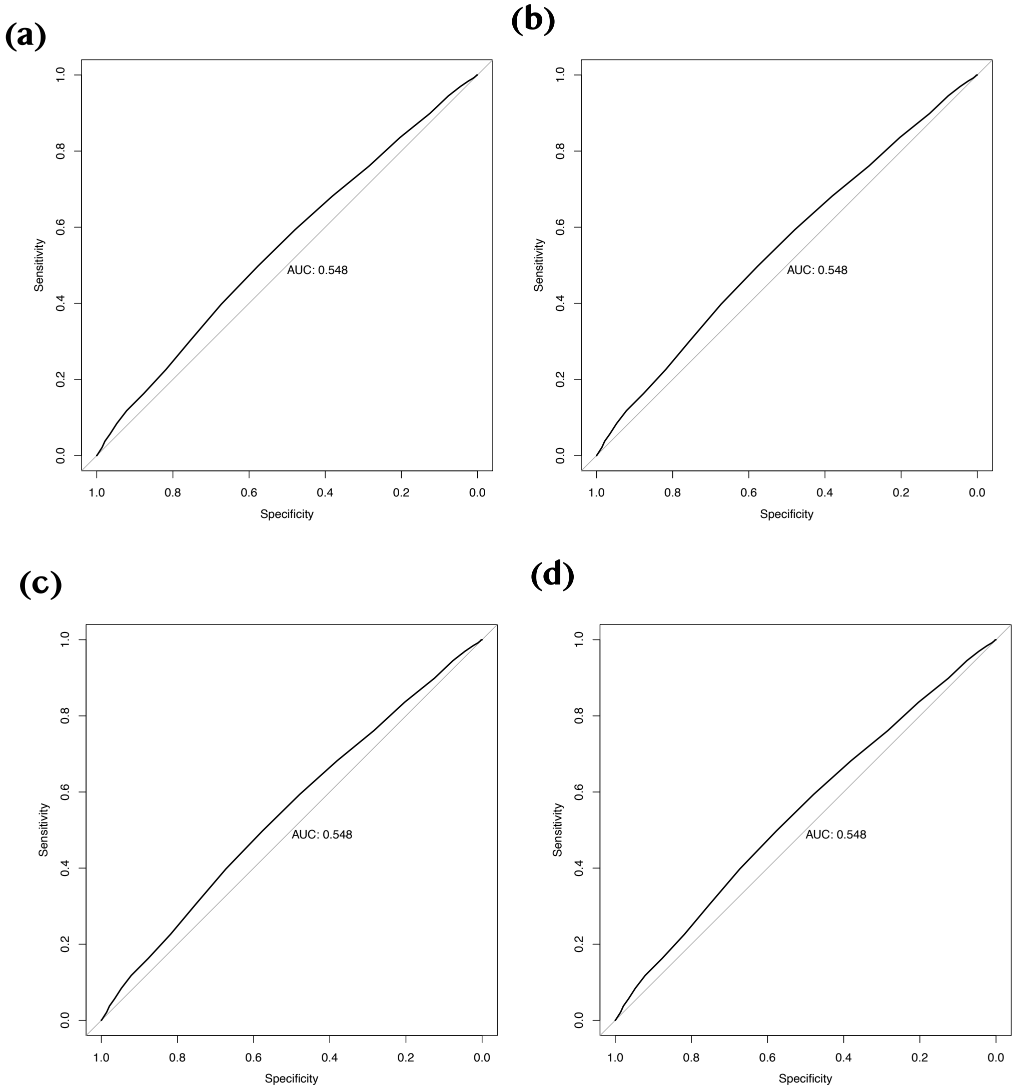

Figure S3. Receiver operating characteristic (ROC) curves evaluating the predictive performance of the discovery-derived polygenic risk score (PRS) in the replication cohort for vitamin D deficiency (25(OH)D < 20 ng/mL). The area under the curve (AUC) was 0.548 ($P = 9.22 \times 10^{-6}$; odds ratio = 0.9995; 95% CI: 0.9993–0.9997), with consistent performance across all *P*-value thresholds used for PRS construction: **(a)** $P < 5{\times}10^{-5}$, **(b)** $P < 5{\times}10^{-6}$, **(c)** $P < 5{\times}10^{-7}$, and **(d)** $P < 5{\times}10^{-8}$.
